# P-2168. Impact of BACT/ALERT® VIRTUO® System on Blood Culture Positivity in Patients with Hematologic Malignancies and Febrile Neutropenia

**DOI:** 10.1093/ofid/ofaf695.2331

**Published:** 2026-01-11

**Authors:** Rachel Ward, Carmen B Smith, Yvonne Burnett, Robin R Chamberland, Tyler Heflin, Sagun Goyal, Christian Gill

**Affiliations:** SSM Health Saint Louis University Hospital, Saint Louis, Missouri; St. Louis College of Pharmacy at UHSP, Saint Louis, Missouri; SSM Health Saint Louis University Hospital, Saint Louis, Missouri; Saint Louis University School of Medicine, St. Louis, Missouri; SSM Health Saint Louis University Hospital, Saint Louis, Missouri; Saint Louis University Hospital, Saint Louis, Missouri; Penn Presbyterian Medical Center, Philadelphia, Pennsylvania

## Abstract

**Background:**

Febrile neutropenia (FN) is an oncologic emergency requiring prompt evaluation for infection and empiric broad spectrum antibiotics; however, blood culture diagnostic yield in FN is low. Increasing culture positivity and reducing time to organism identification can improve antimicrobial use and may reduce risk of subsequent multidrug resistant organisms (MDROs) infections. The BACT/ALERT® VIRTUO®, a fully automated, enclosed blood culture processing system shown to reduce time to positive results, was adopted in January 2022. This retrospective quasi-experimental study aimed to examine the impact of this system on blood culture positivity in patients with FN and hematologic malignancies.

**Figure:**
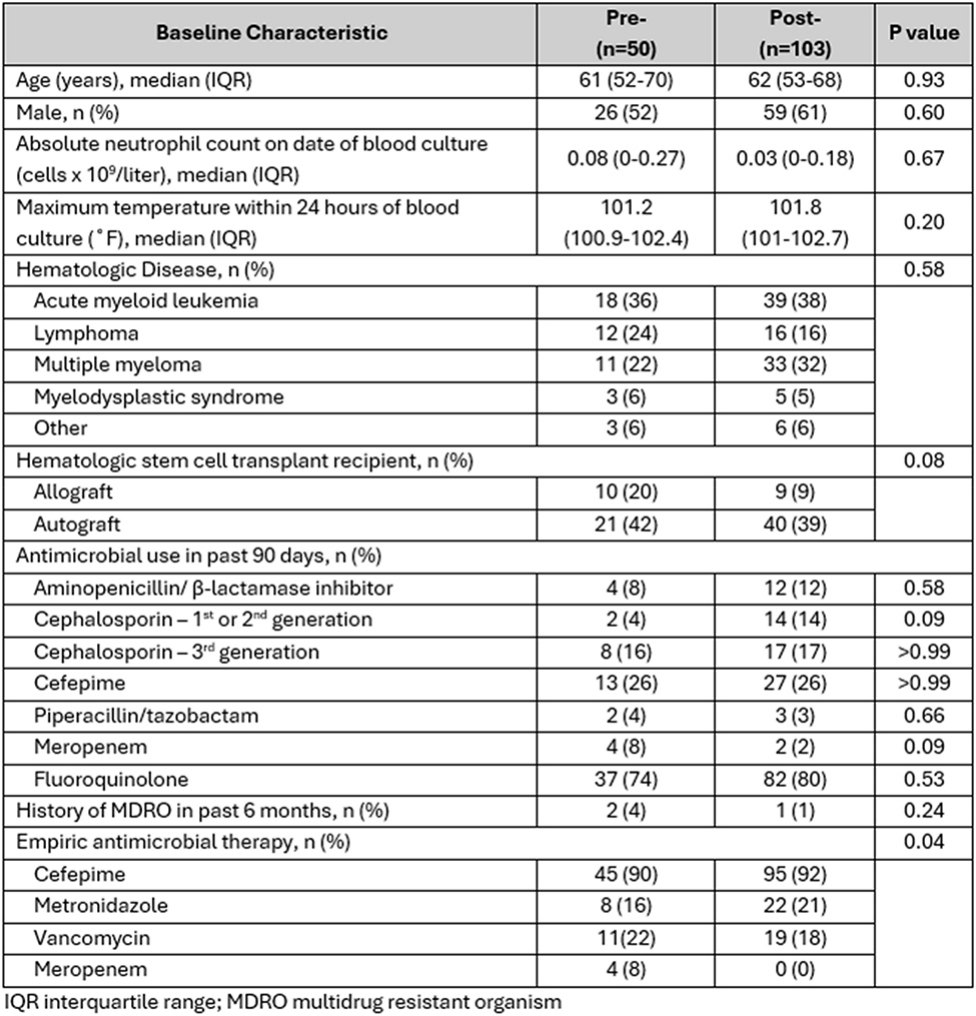

**Figure:**
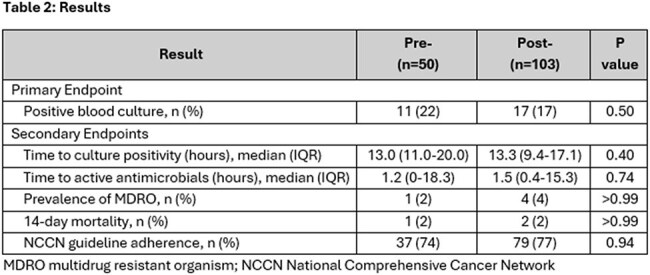

**Methods:**

Blood culture positivity (first set of cultures within 24 hours of FN) in adult patients hospitalized with FN and hematologic malignancy from 1/2021-6/2024 was compared pre- and post- BACT/ALERT® VIRTUO® implementation. Patients were excluded if no blood cultures were collected. Secondary endpoints were time from blood culture collection to culture positivity, time from first fever to active antimicrobial therapy, prevalence of MDROs, 14-day mortality, and adherence to National Comprehensive Cancer Network (NCCN) guideline directed antimicrobial therapy.

**Figure:**
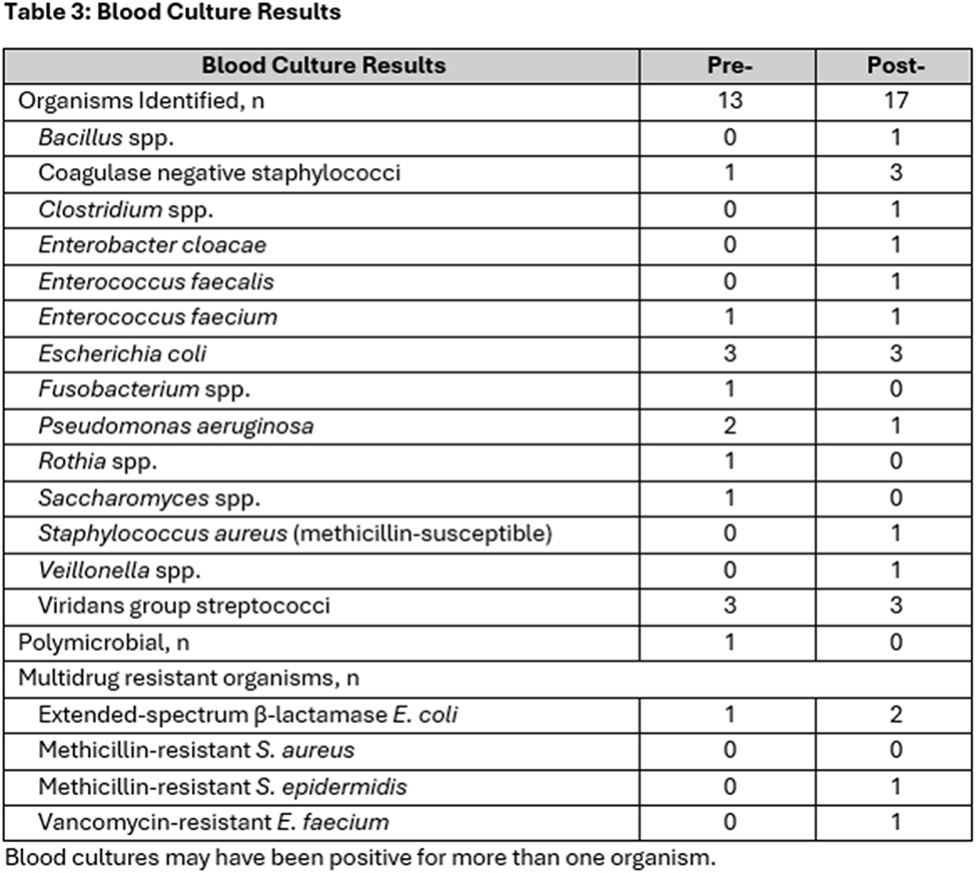

**Results:**

Patients with FN were most often diagnosed with acute myeloid leukemia and multiple myeloma and 52% received hematologic stem cell transplant (Table 1). Culture positivity did not differ significantly pre/post intervention (22% (11/50) vs 17% (17/103); p= 0.504). No differences in median time to culture positivity (13 vs 13 hours), median time to active therapy (1.2 vs 1.5 hours), 14-day mortality (2% (1/50) vs 2% (2/103)) or MDRO prevalence were observed (Table 2), though numerically, more MDRO organisms were identified in the post-implementation group (Table 3). Therapy was concordant with NCCN guidelines in 76% of patients.

**Conclusion:**

The BACT/ALERT® VIRTUO® system did not improve blood culture positivity rates; however, opportunities were identified for improved adherence to NCCN guideline directed therapy and increased monitoring for MDROs in FN.

**Disclosures:**

Yvonne Burnett, PharmD, BCIDP, InflaRx: Honoraria|Melinta Therapeutics: Honoraria Robin R. Chamberland, PhD D(ABMM), bioMerieux: Advisor/Consultant|Pattern Bioscience, Inc.: Advisor/Consultant|Pattern Bioscience, Inc.: Grant/Research Support Christian Gill, PharmD, BCIDP, Cepheid: Grant/Research Support|Cumberland: Grant/Research Support|Entasis: Grant/Research Support|Everest Medicines: Grant/Research Support|Shionogi: Grant/Research Support

